# GDF‐15 is associated with atherosclerosis in adults with transfusion‐dependent beta‐thalassemia

**DOI:** 10.1002/jha2.415

**Published:** 2022-03-22

**Authors:** Alaa Efat, Rana Wahb, Sabry Abd Allah Shoeib, Ashraf Abd ElRaof Dawod, Mohamad Ahmed Abd ElHafez, Essam Ali Abd ElMohsen, Aly Elkholy

**Affiliations:** ^1^ Faculty of Medicine Department of Internal Medicine and Hematology Menoufia University Shebin Al‐Kom Menoufia Egypt; ^2^ Faculty of Medicine Department of Medical Biochemistry Menoufia University Shebin Al‐Kom Menoufia Egypt; ^3^ Department of Hematology and Bone Marrow Transplantation, Maadi Military Forces Medical Complex Maadi, Cairo Governorate Egypt

**Keywords:** CIMT, GDF‐15, transfusion‐dependent beta‐thalassemic adults

## Abstract

Objectives: To study serum growth differentiation factor‐15 (GDF‐15) serum level in β‐thalassemia patients and its relation to carotid intima‐media thickness.

Background: Thalassemia is a common genetic disease resulting in decreased beta‐chains, leading to manifested anemia. It may be subsequently complicated by iron overload, which induces numerous morbidities and even death. Growth differentiation factor‐15 (GDF‐15) is a strong and independent predictor of mortality and disease progression in patients with atherosclerosis alongside with carotid‐intimal media thickness (CIMT).

Patients and methods: This monocentric case‐control study was done on 90 subjects in the period from January 2020 to March 2021. Sixty transfusion‐dependent beta‐thalassemia (TDβT) cases (≥18 years) were selected from the thalassemia clinic of Hematology division at Menoufia University hospitals. We included also 30 sex and age matched healthy as the controls. Routine investigations were done beside. Serum GDF‐15 was measured by ELISA. CIMT was measured by Doppler Ultrasonography.

Results: CIMT on both sides was statistically significant higher in cases (median of 0.08 cm) than in the controls (median of 0.04). GDF‐15 was also significantly higher in cases (median of 1839.89 pg/dl) than in controls (median of 256.14 pg/dl). So, we found that GDF‐15 is a predictor of and associated with atherosclerosis in thalassemic adults (OR = 39.198, *p* value 0.008, 95% CI: 2.576–596.5).

Conclusion: GDF‐ 15 is increased in TDβT. CIMT (as a marker of subclinical atherosclerosis) is increased in these patients alongside with GDF‐15, is a predictor, and associated with atherosclerosis in thalassemic adults.

## 1 INTRODUCTION

Thalassemias are a group of inherited blood disorders caused by decrease or absence of beta‐globin chain synthesis which is reflected with decrease in hemoglobin level; it is mainly inherited as recessive autosomal disorder [1]. Its prevalence is more in Mediterranean countries, the Middle East, Central Asia, the north coast of Africa, and South America. α‐Thalassemia is prevalent in people of Western African and South Asian descent [2]. The beta‐globin chains are encoded by a single gene on chromosome 11 [3].

Insufficient amount of normal structure of globin chains causes imbalance between α and β chains leading to clinical features of the disease [4]. Complications from thalassemia are mainly related to blood transfusion and iron overload that include involvement of the heart, liver, and endocrine glands. Splenomegaly, viral hepatitis infection or human immunodeficiency virus (HIV) infection, venous thrombosis, and osteoporosis may occur also [5]. The relationship between chronic hemolysis with subsequent iron overload, inflammation, and premature atherosclerosis has been documented in hemolytic anemia, especially β‐thalassemia [6]. Carotid intima‐media thickness (CIMT) is a noninvasive method to detect early subclinical atherosclerosis, and it well correlates with vascular injury and severity of coronary artery disease. So, it is a good tool for detecting early atherosclerosis in such patients [7]. Thalassemia intermedia patients usually live longer and, thus, are more prone to complications of atherosclerosis. There is evidence of an increased risk of central ischemia rather than peripheral ischemia in these patients [8]. The growth differentiation factor‐15 (GDF‐15) is a multifactorial cytokine and a member of the transforming growth factor superfamily. Expression of the GDF‐15 gene in cardiomyocytes, vascular smooth muscle cells, and endothelial cells is strongly upregulated in response to oxidative stress, inflammation and tissue injury, while high levels of serum GDF‐15 associate with ineffective erythropoiesis and may reflect a certain type of bone marrow stress or erythroblast apoptosis [9]. In many cardiovascular diseases (such as hypertrophy, heart failure, atherosclerosis, endothelial dysfunction), obesity, insulin resistance, diabetes, and chronic kidney diseases are associated with increased GDF‐15 and linked with the progression and prognosis of the disease condition [10]. In thalssemias and ineffective erythropoiesis, there are high GDF‐15 levels [11].

## 2 PATIENTS AND METHODS

This is a monocentric case‐control study. We included 90 subjects in the period from January 2020 to May 2021. Cases group was 60 transfusion beta‐thalassemia adults (≥18 years); we had selected them from the outpatient thalassemia clinic of Hematology division at Menoufia University hospitals and control group formed of 30 sex and age‐matched healthy subjects.

### 2.1 Ethical approval

Approval of local ethical committee at Menoufia faculty of medicine was obtained under number 121/2021.

Informed consents from all participants were obtained according to local ethical committee guidance.

## 3 METHODS

The diagnosis of beta‐thalassemic adults was based on clinical symptoms and signs, complete blood count (CBC), and hemoglobin electrophoresis.

Following patients were excluded from the study: patients less than 18 years, nontransfusion‐dependent β‐thalassemia adults and other thalassemia variables; patients with other hematologic disorders; as well as patients with heart failure, myopathies, autoimmune or chronic inflammatory disorders.

We define transfusion dependency as the frequency of blood transfusion more than eight transfusions per year [12]. Comprehensive history includedage, sex, smoking, physical and sexual development, number of blood transfusions/ year, iron chelating therapy, which was assessed by reviewing patient report of dose‐taking and the appropriate doses per day, splenectomy, and family history of premature atherosclerotic cardiovascular diseases. Complete clinical examination included cardiac, chest, abdominal, and neurological examination with special emphasis on height, BMI and blood pressure measurement. BMI was calculated with the formula: BMI = weight/height^2^ where weight is measured in kilograms, and height in meters [13].

JNC guidelines of hypertension: normal SBP < 120 mmHg and DBP < 80; prehypertensive SBP 120–139 or DBP 80–89 mmHg; Stage 1 hypertension SBP 140–159 or DBP 90–99 mmHg; Stage 2 hypertension SBP > 160 or DBP > 100 mmHg [14].

Laboratory investigations including complete blood count (CBC), liver function tests (LFTs), kidney function tests (KFTs), virological tests (HCV ab, HBsAg, HBcAb (total), FBS, lipogenic profile (Cholesterol,Triglycerides, LDL and HDL), HOMA IR score, hs CRP, and iron profile (serum iron, serum ferritin, TIBC) were done. A Homeostasis Model Assessment of Insulin Resistance (HOMA‐IR) was used to evaluate insulin resistance and was calculated with the following formula: fasting serum insulin (mU/ml) × fasting plasma glucose (mmol/l)/22.5 [15].

Serum GDF‐15 was estimated by ELISA. The kit of GDF‐15 (Chongqing Biopsies Co., Ltd, China; Catalog No.: BYEK28) was based on standard sandwich enzyme‐linked immune‐sorbent assay technology [16].

We also did pelviabdominal ultrasound and echocardiography to the thalassemic patients participated in the study.

We measured carotid intimal media thickness (CIMT) by Doppler ultrasonography (GE logic E10 United states) and measured at the diastolic phase as the distance between the leading edge of the first and second echogenic lines of the far walls of the distal segment of the common carotid artery, the carotid bifurcation, and the internal carotid artery on both sides, with a duplex ultrasound system with 7.5 MHz scanning frequency in the B‐mode. Measurements were performed 0.5, 1, and 2 cm below and above the bifurcation. Each measurement is an average of three measurements and all measurements were done by a single observer. The mean values of CIMT thickness between patients and controls were compared statistically [17]. The average and maximum CIMT in healthy adults were 0.67 and 0.70 mm, respectively [18].

### 3.1 Statistical analysis

After finishing data collection, we tabulated and statistically analyzed it by an IBM compatible personal computer with SPSS Statistical Package Version 22. Two types of statistics were used: descriptive statistics, for example, number and percent for qualitative data, mean, and standard deviation (SD) for quantitative data and analytic statistics, for example, chi‐squared test (χ^2^) was used to study association between two qualitative variables. Student's *t* test is a test used for comparison between two groups having quantitative normally distributed variables. Mann–Whitney *U* test (nonparametric test) is a test of significance used for comparison between two groups not normally distributed having quantitative variables. Pearson's correlation coefficient test (*r*‐test) is used to study the correlation between two parametric quantitative variables. Spearman's correlation coefficient test (*rs* test) is used to study the correlation between non parametric quantitative variables. Normally distributed data are categorized according to mean and data not normally distributed according to median. Nonsignificant difference if *p* > 0.05, significant difference if *p* < 0.05, and highly significant difference if *p* < 0.001. Test of normality is Kolmogorov–Smirnov. Logistic regression model (univariate and multivariate) analysis was done to find out if GDF‐15 is independent risk factor for increased CIMT (subclinical atherosclerosis) in TDBT. Interpretation of odds ratio (OR): OR = 1 indicates that there is no association between exposure and disease development. OR > 1 indicates that exposure is risky. OR < 1 indicates that exposure is protective. Receiver operator characteristic (ROC) with respective points of maximal accuracy for sensitivity and specificity were generated to determine biomarker performance. Area under the ROC curve (AUROC) measures the accuracy of the test. An area of 1 represents a perfect test; an area of 0.5 represents a worthless test. 0.90–1 = excellent (A), 0.80–0.90 = good (B), 0.70–0.80 = fair (C), 0.06–0.70 = poor (D), 0.50–0.60 = fail (F).

## 4 RESULTS

Results tabulated in Table 1 show that there was no a statistically significant difference between the cases and controls regarding age and sex (*p* value > 0.05). There was a statistically significant difference between them in BMI which was higher in controls by mean of 24.36 ± 2.6 Kg/m^2^ (*p* < 0.05) and highly significant difference in height which was more in controls by mean of 167.7 ± 6.5 cm than in cases by mean of 159.02 ± 8.67 cm (*p* < 0.001). The family history of premature atherosclerosis was higher in cases (63.3%) than in controls (53.3%) with no statistically significant value (*p* > 0.05). There was a highly statistically significant difference regarding Hb level that was lower in cases (mean 7.87 ± 1.01 g/dl) than in controls (mean 12 ± 1.41 g/dl) and platelets were higher in cases (mean 565.47 ± 231.5 × 10^3^/mm^3^) than in controls (mean 305.4 ± 56.43 **×** 10^3^/mm^3^) and TLC that was higher in cases by median of 15 **×** 10^3^/mm^3^ than in controls by median of 7.75 **×** 10^3^/mm^3^ (*p* < 0.001). There was a highly statistically significant difference regarding serum iron that was higher in cases (mean 2.03 ± 0.79 μg/dl) than controls (mean 1.02 ± 0.32 μg/dl), and serum ferritin was higher in cases (median of 2265 ng/ml) than controls (median of 35 ng/ml) (*p* < 0.001). Liver profile was impaired in cases regarding AST, ALT, total bilirubin and direct bilirubin by median of 60, 40, 2.4, and 0.4 mg/dl, respectively, than in controls by median of 26.5, 25, 0.9, and 0.3 mg/dl, respectively, of highly statistically significance (*p* < 0.001). Regarding lipid profile, there was a highly statistically significant difference regarding cholesterol and LDL that were higher in cases by mean of 216.65 ± 49.78 and 179.99 ± 30.93 mg/dl, respectively, triglycerides by median of 123.5, and HDL that was lower in cases by mean of 35.22 ± 6.86 mg/dl than in controls by mean of 54.28 ± 10.26 mg/dl (*p* value was < 0.001). hsCRP was higher in cases by median of 3.35 than in controls by median of 1.73 of highly statistically significance (*p* < 0.001). HOMA‐IR score was higher in controls by median of 0.8 than in cases by median of 0.53 but of no statistically significance (*p* > 0.05). CIMT was more in cases by median of 0.08 cm on right and left sides than in controls by median of 0.04 cm on both sides, of a highly statistically significant value (*p* < 0.001). Regarding GDF‐15, there was a highly statistically significant value that was higher in cases by median of 1839.89 pg/ml than in controls by median of 256.14 pg/ml (*p* < 0.001). Echocardiography showed a highly statistically significant difference between cases and controls in diastolic dysfunction that was higher in cases by percentage of 56.7% while 3.3% of cases had systolic dysfunction. Pulmonary hypertension was more in cases by mean of 30.12 ± 9.7 mmHg than in controls by mean of 15.132 ± 2.9 mmHg of highly statistically significance as shown in Table 1.

**TABLE 1:** Comparison between patients and controls regarding demographic, clinical, and hematological variables (*n* = 90)

Variable	Cases	Controls	*p* Value
	(*n* = 60)	(*n* = 30)	
**Age, years**			
Mean ± SD	27.17 ± 5.75	28 ± 5.81	0.520
**Sex, no. (%)**			
Male	23(38.3%)	15(50%)	0.291
**Height, cm**			
Mean ± SD	159.02 ± 8.67	167.7 ± 6.5	<0.001
**BMI, kg/m^2^**			
Mean ± SD	23.05 ± 2.59	24.36 ± 2.6	0.026
**Smoking, no. (%)**			
Yes	10 (16.7%)	2 (6.7%)	0.188
**Hypertension**			
Yes	6 (10%)	0 (0%)	0.073
**Family history of premature atherosclerosis**			
Yes	38 (63.3%)	16 (53.3%)	0.361
**Hemoglobin, g/dl**			
Mean ± SD	7.87 ± 1.01	12 ± 1.41	<0.001
**TLC, ×10^3^/mm^3^**			
Median	15	7.75	<0.001
Range	5.3–72	4–12	
**Platelets, ×10^3^/mm^3^**			
Mean ± SD	565.47 ± 231.5	305.4 ± 56.43	<0.001
**Iron, μg/dl**			
Mean ± SD	2.03 ± 0.79	1.02 ± 0.32	<0.001
**S. Ferritin, ng/ml**			
Median	2265	35	<0.001
Range	150–10441	19–37	
**Transferrin saturation, %**			
Median	75.5	30.19	<0.001
Range	21–119	10–48	
**PC, %**			
Mean ± SD	77.38 ± 9.29	85.83 ± 7.43	<0.001
**AST, U/L**			
Median	60	26.5	<0.001
Range	18–278	17–40	
**ALT, U/L**			
Median	40	25	<0.001
Range	11–225	15–50	
**Total bilirubin, mg/dl**			
Median	2.4	0.9	<0.001
Range	1.2–9	0.5–1.2	
**direct bilirubin, mg/dl**			
Median	0.4	0.3	<0.001
Range	0.2–1.8	0.1–0.8	
**Indirect bilirubin, mg/dl**			
Median	1.9	0.6	<0.001
Range	1–7.2	0.4	
**Creatinine, mg/dl**			
Median	0.6	0.6	0.775
Range	0.3–1.3	0.3–0.9	
**Ca, mg/dl**			
Mean ± SD	8.01 ± 0.97	9.04 ± 0.54	<0.001
**Albumin, g/dl**			
Mean ± SD	3.73 ± 0.3	4.22 ± 0.51	<0.001
**UA, mg/dl**			
Mean ± SD	5.52 ± 1.37	4.5 ± 0.82	<0.001
**Cholesterol, mg/dl**			
Mean ± SD	216.65 ± 49.78	103.73 ± 14.86	<0.001
**Triglycerides, mg/dl**			
Median	123.5	92.5	0.001
Range	49–337	51–139	
**LDL, mg/dl**			
Mean ± SD	179.99 ± 30.93	85.19 ± 12.59	<0.001
**HDL, mg/dl**			
Mean ± SD	35.22 ± 6.86	54.28 ± 10.26	<0.001
**FBS, mg/dl**			
Mean ± SD	92.2 ± 31.81	87.97 ± 7.13	0.333
**hsCPR**			
Median	3.35	1.73	0.001
Range	0.23–35.4	3–3.39	
**HOMA—IR, mg/dl**			
Median	0.54	0.8	0.137
Range	0–2.87	0.4–1	
**HBc Ag**			
Positive	3(5%)	0(0%)	0.111
**HCV Ab**			
Positive	17(28.3%)	0(0%)	0.001
**Left CIMT, cm**			
Median	0.08	0.04	<0.001
Range	0.05–0.2	0.03–0.05	
**Right CIMT, cm**			
Median	0.08	0.04	<0.001
Range	0.04–0.2	0.03–0.05	
**GDF‐15, pg/ml**			
Median	1839.89	256.14	<0.001
Range	1100–3641	108–495.28	
**Abdominal ultrasound**			
Splenectomy	42 (70%)	0 (0%)	<0.001
Splenomegaly	18 (30%)	0 (0%)	
**Systolic dysfunction**			
Yes	2 (3.3%)	0 (0%)	0.551
No	58 (96.7%)	30 (100%)	
**Diastolic dysfunction**			
Yes	34 (56.7%)	0 (0%)	<0.001
No	26 (43.3%)	30 (100%)	
**Pulmonary hypertension**			
Mean ± SD	30.12 ± 9.7	15.132 ± 2.9	<0.001

*Note: p* < 0.001 is statistically highly significant and *p* < 0.05 is statistically significant.

^@^Spearman's correlation.

TLC : total leucocyte count; ALT: alanine aminotransferase; Hs CRP: high‐sensitivity C‐reactive protein; HDL: high‐density lipoproteins; CIMT: carotid intimal media thickness; HOMA‐IR score: homeostatic model assessment for insulin resistance; AST: aspartate aminotransferase; PC: prothrombin concentration; LDL: low‐density lipoproteins; FBS: fasting blood sugar; UA: uric acid.

From Table 2 we have a correlation between CIMT and other variables in thalassemic patients. A positive correlation was found between CIMT and serum ferritin and iron levels of highly statistically significance (*p* < 0.001). A positive correlation was present with uric acid and serum calcium of statistically significant value (*p* < 0.05). Regarding lipid profile, a positive correlation with highly statistically significance with LDL and cholesterol was present (*p* < 0.001). A negative correlation with HDL of a highly statistically significant value was present (*p* < 0.001). Regarding GDF‐15, there was a positive correlation between CIMT and it with a highly statistically significant value (*p* < 0.001). A positive correlation was present with history of blood transfusion dependency of statistically significance (*p* < 0.05). Negative correlation was present between CIMT and regular ICA intake of statistically significance (*p* < 0.05).

**TABLE 2:** Correlation between carotid artery intima media thickness and other variables of the patients

Variables	Right CIMT *p* Value		Left CIMT *p* Value	
	*rs*		*rs*	
**Age (years)**	0.037	0.780	0.127	0.335
**Smoking**	0.178	0.173	0.103	0.435
**FH of premature CAD**	0.006	0.963	0.031	0.817
**Hypertension**	0.084	0.525	0.056	0.673
**BMI (kg/m^2^)**	0.013	0.919	0.14	0.919
**Hemoglobin (g/dl)**	0.045	0.733	0.108	0.441
**TLC (×10^3^/mm^3^)**	0.225	0.03*	0.229	0.03*
**Platelets (×10^3^/mm^3^**)	–0.053	0.686	–0.013	0.323
**Iron (μg/dl)**	0.252	0.001*	0.367	<0.001*
**S. Ferritin (ng/ml)**	0.27	0.001	0.427	<0.001*
**AST (U/ml)**	–0.074	0.575	–0.079	0.547
**ALT (U/ml)**	0.106	0.420	0.121	0.355
**Total bilirubin (mg/dl)**	0.013	0.919	0.016	0.93
**direct bilirubin (mg/dl)**	–0.070	0.597	–0.037	0.780
**indirect bilirubin (mg/dl)**	0.012	0.930	0.007	0.957
**Ca (mg/dl)**	0.391	0.003*	0.371	0.004*
**UA (mg/dl)**	0.186	0.155	0.109	0.409
**Cholesterol (mg/dl)**	0.403	<0.001	0.328	0.011*
**Triglyceride (mg/dl)**	0.006	0.964	0.036	0.785
**LDL (mg/dl)**	0.335	0.001*	0.667	<0.001*
**HDL (mg/dl)**	–0.306	0.003*	–0.491	<0.001*
**FBS (mg/dl)**	0.139	0.294	0.093	0.484
**HOMA‐IR (mg/dl)**	0.117	0.372	0.06	0.645
**hs CPR**	0.134	0.306	0.052	0.695
**GDF15 (pg/ml)**	0.903	<0.001**	0.817	<0.001**
**Blood transfusion**	0.321	0.012	0.326	0.011
**Regular ICA**	–0.321	0.012	–0.350	0.006

*Note: rs* is Spearman's correlation.

* significant.

** highly significant.

Correlation between GDF‐15 and other variables in beta‐thalassemic patients is shown in Table 3. Regarding lipid profile, there was a positive correlation between GDF‐15 and cholesterol of statistically significance (*p* < 0.05). A positive correlation with hs CRP with highly statistically significance (*p* < 0.001) was present. A positive correlation between GDF‐15 and history of blood transfusion dependency of statistically significant value (*p* < 0.05) and negative with history of ICA therapy of statistically significance was present. No significant correlation was present between GDF‐15 and age, BMI, hypertension, smoking and family history of atherosclerosis, hematological parameters, serum ferritin levels, serum iron, triglycerides, LDL, HDL, FBS, and HOMA‐IR score.

**TABLE 3:** Correlation between GDF15 level and other variables of the patients

Variables	GDF15	
	*rs*	*p* Value
**Age (years)**	0.045	0.732
**Smoking**	0.063	0.631
**Family history of premature CAD**	0.11	0.399
**Hypertension**	0.061	0.644
**BMI (Kg/m^2^**	0.073	0.577
**Hemoglobin (gm/dl)**	0.018	0.890
**TLC (×10^3^/mm^3)^**	–0.064	0.629
**Platelets count (×10^3^/mm^3)^**	–0.090	0.492
**Iron (μg/dl)**	0.011	0.931
**S. Ferritin (ng/ml)**	0.047	0.722
**AST(U/L)**	–0.157	0.232
**ALT(U/L)**	0.059	0.653
**Total bilirubin (mg/dl)**	0.141	0.282
**Direct bilirubin (mg/dl)**	0.148	0.259
**Indirect bilirubin (mg/dl)**	0.130	0.323
**Creatinine (mg/dl)**	0.063	0.136
**Ca (mg/dl)**	–0.066	0.619
**UA (mg/dl)**	0.133	0.311
**Cholesterol (mg/dl)**	0.365	0.004*
**Triglycerides (mg/dl)**	0.033	0.801
**LDL (mg/dl)**	0.083	0.530
**HDL (mg/dl)**	0.143	0.274
**FBS (mg/dl)**	0.207	0.116
**HOMA—IR (mg/dl)**	0.229	0.078
**hs CPR**	0.098	0.456
**History of blood transfusion dependency**	0.315	0.014
**Regular ICA therapy**	–0.340	0.008

* significant.

**FIGURE 1:**
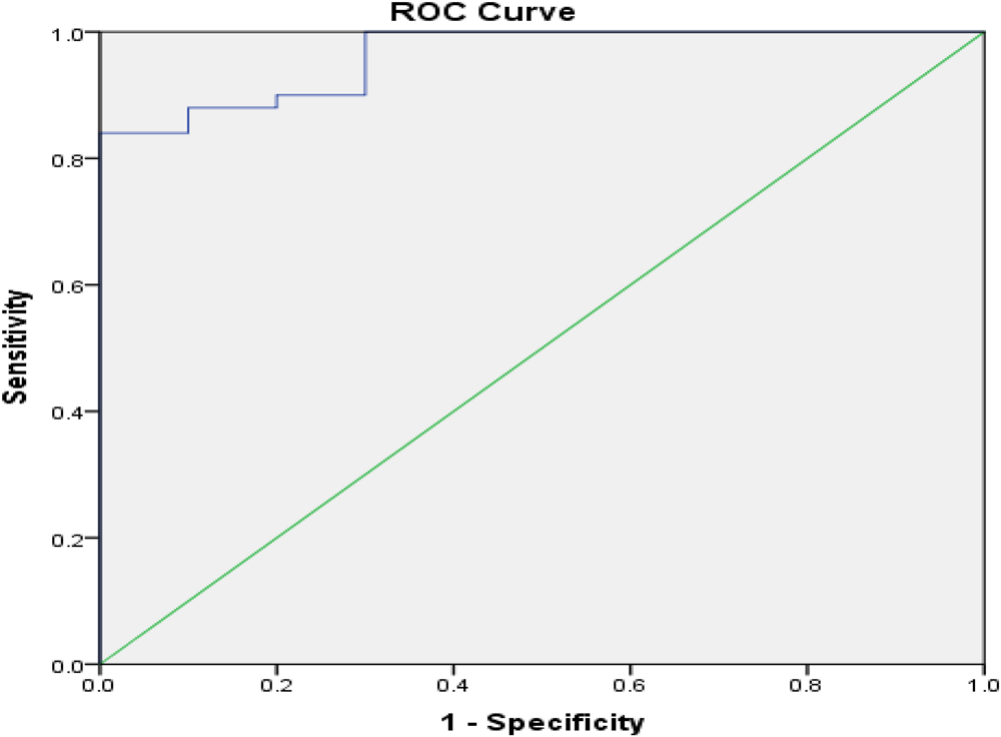
Receiver operating characteristic curve (ROC) analysis of the optimal cutoff of CIMT and GDF15 levels. Blood GDF‐15 levels classified into three categories: normal (<1200 pg/ml), moderately elevated (1200–1800 pg/ml), and highly elevated (>1800 pg/ml) [35]

Receiver operating characteristic curve analysis of the optimal cutoff of GDF‐15 for prediction of atherosclerosis was done in thalassemic patients as in Table 4. The serum GDF‐15 to CIMT ratio as a predictor of atherosclerosis with cut off value of GDF‐15 (≥1446.01 pg/dl) is highly statistically significant (*p* < 0.001) and area under curve (AUC) is 0.962 of specificity 90% and sensitivity 88% as shown in Figure 1.

**TABLE 4:** Receiver operating characteristic curve analysis of the optimal cutoff of CIMT and GDF15 levels

Cutoff point	AUC	Sensitivity%	Specificity%	*p* Value	95%CI(lower–upper)
GDF‐15 level					
≥1446.01	0.962	88%	90%	<0.001**	0.915–1

** highly significant.

Table 5 shows univariate regression analysis of factors that were independently associated with increased CIMT. It was performed on a number of predictors including age, sex, BMI, smoking, history of premature CAD, hypertension, hemoglobin level, ferritin, transferrin saturation, insulin resistance (HOMA‐IR score), lipid profile (cholesterol, triglycerides, LDL, and HDL), hs CRP, history of splenectomy, dependency on blood transfusion, regular ICA therapy, and GDF‐15 level. It was found that the most important factor is the GDF‐15 (odds ratio: 58.5, 95% CI: 11.919–278.120, *p* < 0.001) of highly statistically significance, followed by smoking and blood transfusion dependency (odds ratio: 5.524 and 4.767, 95% CI: 1.063–28.708, 1.171–19.396, *p* < 0.05, respectively) of statistically significance, then, serum ferritin, regular ICT therapy, cholesterol, and HDL (odds ratio: 3.519, 0.1, 3.077, and 0.289, 95% CI: 1.209–10.240, 0.01–0.742, 1.064–8.899, and 0.1–0.834, *p* < 0.05) respectively of statistically significant, followed by LDL, age, sex, hypertension, BMI, Hb level, history of premature CAD, triglycerides, hs CRP, HOMA‐IR score, and history of splenectomy of no statistically significance (*p* > 0.05).

**TABLE 5:** Univariate analysis for variables associated with CIMT in thalassemic patients (*n* = 60)

Predictors	Odds ratio	95% CI (lower–upper)	*p* Value
Age (≥27 years)	1.825	0.565–5.895	0.314
Sex (male)	1.706	0.597–4.876	0.319
Smoking (yes)	5.524	1.063–28.708	0.042*
Family history of premature CAD (yes)	0.9	0.315–2.574	0.844
Hypertension (yes)	1.077	0.199–5.819	0.931
BMI (≥23 kg/m^2^)	0.867	0.314–2.393	0.782
Hb (>7.87 g/dl)	1.495	0.540–4.136	0.439
Serum ferritin (≥2265)	3.519	1.209–10.240	0.021*
Transferrin saturation % (≥75)	1.962	0.702–5.479	0.199
Cholesterol (≥216.65)	3.077	1.064–8.899	0.038*
Triglycerides (≥123.5)	1.143	0.415–3.148	0.796
LDL (≥179.99)	3.594	1.147–11.256	0.28*
HDL (≥35.22)	0.289	0.1–0.834	0.022*
hs CRP (≥3.35)	0.510	0.183–1.424	0.199
HOMA‐IR (≥0.54)	1.962	0.702–5.479	0.199
Spleen (splenectomy)	0.909	0.301–2.744	0.866
Blood transfusion (monthly)	4.767	1.171–19.396	0.029*
Regular ICA therapy	0.1	0.01–0.742	0.026*
GDF‐15 (pg/ml) (≥1839.89)	58.5	11.919–278.120	<0.001**

* significant.

** highly significant.

Table 6 shows multivariate logistic regression analysis of variables associated with increasing CIMT (subclinical atherosclerosis). GDF‐15 is the most important predictor associated with increasing CIMT in thalassemic patients (OR 62.143, 95% CI: 5.780–66.166; *p* < 0.001) of highly statistically significant value. Followed by serum ferritin (OR was 4.312, 95% CI: 1.116–16.656, *p* = 0.034) of statistically significance, then LDL and HDL (OR was 3.875, 0.74, 95% CI: 0.929–16.168, 0.591–0.832, *p* = 0.05 and = 0.01, respectively) followed by blood transfusion dependency, regular ICA therapy, smoking, and cholesterol of no statistically significance.

**TABLE 6:** multivariate analysis for variables significantly associated with increased CIMT in thalassemic patients (*n* = 60)

Predictors	Odds ratio	95% CI (lower‐upper)	*p* Value
Smoking (yes)	2.734	0.440–16.990	0.281
Serum ferritin (≥2265)	4.312	1.116–16.656	0.034*
Cholesterol (≥216.65)	3.114	0.795–12.199	0.103
LDL (≥179.99)	3.875	0.929–16.168	0.063
HDL (≥35.22)	0.74	0.591–0.832	0.013*
Blood transfusion dependency (monthly)	2.771	0.455–16.856	0.269
Regular ICA therapy (yes)	0.36	0.017–4.362	0.174
GDF15 (pg/ml) (≥1839.89)	62.143	5.780–66.166	0.001*

* significant.

## 5 DISCUSSION

High‐resolution ultrasound is a method for detecting structural and functional atherosclerotic changes in the arterial wall. Intima‐medial thickness (IMT) is a measure of the combined thickness of intima and media layers of carotid artery that is assessed by B‐mode ultrasound. Increased CIMT is a structural marker representative of subclinical and asymptomatic atherosclerotic vascular diseases [19]. The aim of our study is to evaluate GDF‐15 serum levels and CIMT (as a biomarker for subclinical atherosclerosis) in a cohort of transfusion‐dependent beta‐thalassemic adults to explore their possible correlations with clinical, hematological, and laboratory variables and to reveal the association between risk factors and atherosclerosis. The study showed that age varies with a mean of 27.17 ± 5.75 years. There was female predominance by percentage of 61.6%. There was a statistically significant difference between cases and controls in BMI that was higher in controls (*p* < 0.05) and a highly significant difference in height that was more in controls by mean of 167.7 ± 6.5 cm than in cases by mean of 159.02 ± 8.6.7 cm (*p* < 0.001), and this is similar to studies of Abd Elsamei et al. [20] and Ghazala et al. [21]. There was a highly statistically significant difference regarding Hb levels that were lower in cases (mean 7.87 ± 1.01 g/dl) than in controls (mean 12 ± 1.41 g/dl) and platelets that were higher in cases (mean 565.47 ± 231.5×10^3^/mm^3^) than in controls (mean 305.4 ± 56.43×10^3^/mm^3^). This is similar with mean Hb levels of 10.73 ± 1.67 g/dl [22]. Iron overload is an unavoidable complication suffered by thalassemia major patients as a consequence of excessive number of blood transfusions. There was a highly statistically significant difference regarding serum iron that was higher in cases (mean 2.03 ± 0.79 μg/dl) than in controls (mean 1.02 ± 0.32 μg/dl), serum ferritin that was higher in cases (median of 2265 ng/ml) than in controls (median of 35) (*p* < 0.001) which is similar to Riaz et al. [23] study that showed higher ferritin levels by mean of 4236.5 ± 2378.3 ng/ml and Mishra et al. [24] with serum ferritin median levels of 2767.5 ng/ml in thalassemic patients. Also Sabry et al. [25] study showed that (86%) of the thalassemic patients have ferritin levels more than 1000. Lipid profile showed a highly statistically significant value regarding cholesterol and LDL that were higher in cases (*p* < 0.001) as in Sherief et al. [26] that showed high lipogenic profile in patients. Endothelial dysfunction and arterial thickness are risk factors for the development of atherosclerosis. In our study, CIMT was more in cases by median of 0.08 cm on both sides than in controls (median of 0.04 cm) of highly statistically significant value (*p* < 0.001). This is consistent with Sherief et al., which showed increased CIMT on both sides by mean of 0.62 ± 0.20 mm on right side and 0.66 ± 0.17 mm on left side. GDF‐15, a member of the TGF super family, was elevated in beta‐thalassemia and contributes to hepcidin suppression this is consistent with our study, as it was higher in cases by median of 1839.89 pg/ml than in controls (median of 256.14 pg/ml) of highly statistically significant value (*p* < 0.001). And this is similar to Athiyarath et al. [27] who showed increased GDF‐15 in thalassemic patients with median of 6059.87 pg/ml. Cardiac involvement is an important complication of thalassemia major. It was higher in cases regarding diastolic dysfunction (56.7%) and systolic dysfunction (3.3%) and pulmonary hypertension that were high in cases by mean of 30.12 ± 9.7 mmHg than in controls (*p* < 0.001). This is similar to Khalid et al. [28] study in Egypt that showed increased incidence of heart failure and pulmonary hypertension in thalassemic patients. Results show a positive correlation between CIMT, age, and BMI but of no statistically significance as in study of Munckhof et al. [29] that showed that CIMT increases with age and BMI carrying evidence of increased CV diseases incidence with age. There was a positive correlation between CIMT with serum iron and serum ferritin levels of highly statistically significance (*p* < 0.001). This is matched with Sherief et al. who showed a positive correlation between them. There is a positive correlation with FBS and was present between CIMT, cholesterol, LDL, and triglycerides and a negative correlation with HDL of a highly statistically significance is found in study of El‐Masry et al. [30] regarding insulin resistance and CIMT. There is positive correlation with FBS and HOMA‐IR score of no statistically significance and a positive one with hs CRP with highly statistically significance (*p* < 0.001). Also, study of de Lima Sanches et al. showed a positive correlation between insulin resistance and CIMT and increased HOMA‐IR score [31]. There was a positive correlation between GDF‐15 and CIMT of highly statistically significance (*p* < 0.001), which is consistent with Sherief et al. that showed increased GDF‐15 with CIMT in thalassemic patients. A positive correlation was present in our study between GDF‐15 and age as in Liua et al. that showed a positive correlation between them and GDF ‐15 levels increase with age [32]. There is a positive correlation between GDF‐15, hs CRP, and HOMA‐IR score as in the study of Roy et al. that showed the same results and identified that GDF15 levels as marker of T2DM in obese patients [33]. Multiple logistic regression analysis of factors that might independently be associated with CIMT was performed. It was found that the most important predictor with CIMT is GDF‐I5 (OR = 39.198, *p* = 0.008, 95% CI: 2.576–596.5) of statistically significance. A study of He et al. that shows that increased circulating GDF15 levels were closely associated with cardiovascular diseases and were shown to be a strong marker of disease progression in patients with atherosclerosis [34].

## 6 CONCLUSION

Subclinical atherosclerosis was documented among Egyptian transfusion‐dependent beta‐thalassemic adults. This is evaluated by measurement of CIMT (as a biomarker of subclinical atherosclerosis) by ultrasonography and was positively correlated with dyslipidemia and elevated serum GDF‐15, which is detected by ELISA. So, GDF‐15 is associated with atherosclerosis in adults with transfusion dependable beta‐thalassemia.

## CONFLICT OF INTEREST

The authors disclose no conflict of interest.

## FUNDING

None.

## AUTHOR CONTRIBUTIONS

Alaa Efat and Rana Wahb wrote the manuscript and analyzed the data. Aly Elkholy and Mohamed Abdelhafez performed data collection and manuscript preparation. Ashraf Dawod performed laboratory studies and analysis. Sabry Shoeib and Essam Abdelmohsen were responsible for selection and follow‐up of patients. All authors revised the study and reviewed the article.

## ETHICS STATEMENT

Approval of local ethical committee at Menoufia faculty of medicine was obtained under number 121/2021. Informed consents from all participants were obtained according to local ethical committee guidance.
